# Laboratory Investigations of the Leica RTC360 Laser Scanner—Distance Measuring Performance

**DOI:** 10.3390/s24123742

**Published:** 2024-06-08

**Authors:** Peter Bauer, Helmut Woschitz

**Affiliations:** Institute of Engineering Geodesy and Measurement Systems, Graz University of Technology, A-8010 Graz, Austria; peter.bauer@tugraz.at

**Keywords:** terrestrial laser scanning, Leica RTC360, distance performance, horizontal comparator, laboratory investigations

## Abstract

A Leica RTC360 laser scanner was investigated using a linear horizontal comparator system with four targets of different reflectance. Several thousand panorama scans were conducted along the 30 m long comparator, basically in 40 mm steps. For a selected target, more detailed investigations were carried out with a 2 mm step width for a 2 m wide section. The absolute offset between the scanner and the relative interferometer measurements was determined with a calibrated total station. The investigations revealed several systematic effects like an offset in the distance measurement of about 1.3 mm. Furthermore, sections with stochastic behavior as well as sections with pseudo-cyclic parts were observed, depending on the reflectance of the target. The deterministic sections showed curved and striped patterns with some discontinuities of about 2 mm at 20 m, resulting in a saw-tooth like pattern along the distances. Within all the experiments, the distance deviations were below the manufacturer specifications of the 3D point accuracy. However, it was demonstrated that the distance measurements had clear systematic components. In using these new findings, the specification of the measurement “noise” in the data sheet has to be seen as critical.

## 1. Introduction

Ensuring data quality is a crucial part of data acquisition, especially for critical applications, like deformation monitoring. However, in real life projects, it is becoming somehow more complicated to evaluate and state the actual measurement accuracy. Modern surveying instruments are multi-sensor systems and use complex software algorithms in order to provide optimal results under different environmental conditions. Especially in TLS, the derived point coordinates are not only dependent on the raw-angle and distance measurements but also affected by various internal corrections. Furthermore, data sets are usually preprocessed (e.g., filtered) in the instrument, upon data collection, and further postprocessed in the registration software. Thus, it is even harder to state an instrument-dependent accuracy information for the point cloud, because the effects of hardware and software can hardly be distinguished. Therefore, laser scanners are mostly treated as a black-box system in practical examples, because a detailed understanding of the measuring technology is often missing or the complex internal processes are not communicated to the user.

Every well-known manufacturer provides appropriate data sheets for their instruments (see, for instance, [1,2,3]). However, specifying the uncertainty of laser scanners is not an easy task due to different surface properties, geometric and registration issues, the validity of the internal calibration parameters for the current circumstances, and atmospheric refraction. Therefore, manufacturers often specify only measurement precision for certain targets, in laboratory conditions and for selected distances with specific surface properties. In particular, for a rather large TLS user group with only little or without metrological background, this becomes a problem if the specifications of the manufacturers are misunderstood or misinterpreted. Dazzled by this, the accuracy expectations for real world applications are often too ambitious. This leads to problematic results, especially in high-accuracy applications like deformation monitoring, but this also influences the documentation and preservation of evidences (e.g., heritage documentation, monument preservation, or crime scene investigations).

There is a clear need for a better understanding of laser scanner devices to support more realistic accuracy expectations. This can be seen by the amount of research that is currently being conducted on TLS. Therefore, a lot of the literature and several PhD theses are available that have performed accuracy investigations and system evaluations of different scanners and contributed significantly to the common understanding of TLS, including on the calibration of 3D laser scanners [4], on the stochastic properties of point clouds [5], on 2D deformation monitoring with laser scanners [6] or on the surface determination with laser scanners [7], among many others.

This need has also been the catalyst for the so-called “Collector Project” [8], which was organized by the Society for Calibration of Geodetic Devices (SCGD) [9]. Different research groups were invited to determine the geometry of a rather simple object (i.e., a large and almost flat concrete wall) using different types of TLS, but with the same processing workflow. The wall was scanned from three distinct set-up locations (different scanning distances and incidence angles). Comparing the results should reveal the measuring capabilities of the different instruments used. We participated in this project with our RTC360 from Leica Geosystems (Heerbrugg, Switzerland). and found systematic effects in the lower millimetre range inherent in the measurements, as other RTC360 participants (ETH Zürich).

**Figure 1:**
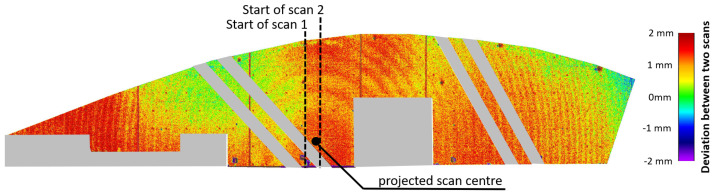
Deviations of two scans performed from different set-up locations of the Bonn reference wall, with the TU Graz RTC360, as part of the Collector Project (inhomogeneous areas are colored gray).

Figure 1 shows the deviations between two scans, both almost in the same orthogonal line to the wall. Besides minor registration misalignments, other systematic effects, which show a cyclic-like pattern with a magnitude of about 1 mm, are visible around the projected scan center. This error pattern was unexpected for the RTC360 and is thus new to the community, which motivated follow-up investigations in different fields by the contributing institutes.

Thus, in order to contribute to the ongoing discussions in the geodetic community about the wave form digitizing (WFD) technology [10] used by the Leica RTC360, we designed some dedicated experiments in our laboratory, mainly using the 30 m long horizontal comparator bench. The main findings of this investigations will be presented and discussed in this paper and might help to obtain a better understanding of the technology used and accuracy expectations. Additionally, to support more detailed studies by other institutions, we made the laboratory data, which are presented in this paper, available to the community as open data [11].

## 2. The Leica RTC360 Laser Scanner

**Figure 2:**
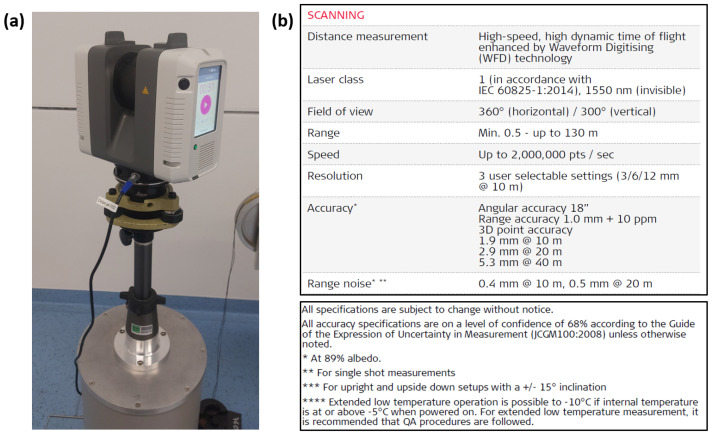
(**a**) Image of the Leica RTC360 in the Laboratory and (**b**) a collage of two screenshots of the manufacturer’s data sheet [3].

The Leica RTC 360 (Figure 2a) is a medium-range panorama scanner with a working range from 0.3 m to 130 m. Three different spatial resolutions (3/6/12 mm @ 10 m) are available, affecting the sampling of the object, but not the accuracy of the acquired point cloud data. The distance measuring accuracy is specified in the data sheet as 1 mm + 10 ppm, with a level of confidence of 68%, for single-shot measurements to a surface with 89% reflectance [3]. Furthermore, the range noise is specified as 0.4 mm at a distance of 10 m and 0.5 mm at 20 m ([3], Figure 2b).

The technology used for the distance measurement is described also as *“[…] time-of-flight enhanced by Waveform Digitising (WFD) technology […]”* in [12]. Further, the WFD technology is (briefly) described in the Leica white paper [10]. More details about the RTC360 are given by Biasion et al. [13], but regarding the electronic distance measurement (EDM), they only note that *“[…] two pulses are modulated to the emitted laser beam […]”*. The use of two pulses is among the key features of the RTC360; they differ in intensity (ratio of about 1:7) and are shifted by 75 ns to each other. The pulse with the higher energy is used for measuring the distances to surfaces of low reflectance, and the lower energy pulse is used for surfaces of higher reflectance. The strong pulse is the first of the two pulses and saturates the receiver diode for high-reflective surfaces, which is why the weak pulse is predominant in the distance measurements [13].

Some more details, relevant for the investigations shown in this paper, are provided in the user manual [12]: The scanning laser has a wavelength of 1550 nm, a beam diameter (1/$e^{2}$) of 6 mm at the front window of the scanner, and a beam divergence of 0.5 mrad (1/$e^{2}$, full angle). The pulse has a duration of 0.5 ns, the mirror rotates with 100 Hz, and 2 million points are measured per second in the 3 mm mode.

## 3. Methodology

### 3.1. Related Research

Although a lot of research is currently going on in the field of laser scanning, detailed investigations, focusing solely on the distance measuring capabilities of specific systems, are rare. One of the main reasons might be that well-established, rather complex, laboratory facilities are required, which are only accessible to a limited group of researchers. Especially if the laboratory facility has to be rented, this comes with high costs and might be usually conducted by manufacturers for the development of prototypes. However, these data sets are often not available to the community.

Examples of detailed laboratory investigations of TLS on comparator benches can be seen for the laser scanner ZF5006i (from Zoller+Fröhlich, Wangen im Allgäu, Germany) [14] or in ongoing investigations carried out at ETH Zürich by the group of Prof. A. Wieser for the Faro Focus X laser scanner (A. Wieser, personal communication). These references show that such investigations and the interpretations of the results are highly sophisticated.

One of the very few investigations that provide an independent impression of the distance performance of the RTC360 was conducted at HCU Hamburg by the group of Prof. T. Kersten [15]. RTC360 measurements were carried out on a scanning target that was shifted on their 20 m long linear rail system. The derived distances were compared to reference values (gathered with a Leica TS60 total station). However, the shown distance resolution (1 m steps) and the precision of the reference system do not allow for more detailed studies on the scanner’s distance performance. Another investigation that is important for this work (in order to find reliable points on an EDM target) is the determination of the resolution capabilities by Jost et al. [16]. The spot size provides information about the ability of a TLS to resolve two adjacent objects in the line of sight. For these investigations, a 1.25 × 1.25 m^2^ large Böhler Star was used to determine the spatial resolution of several TLS at different distances. For the RTC360, they determined a symmetrical resolution performance of about 9 mm at a 10 m distance and about 22 mm at a 30 m distance, which fits well with the manufacturer specifications of the laser beam.

### 3.2. Concept for Testing the Distance Measurement Performance of the RTC360

Comprehensive system investigations evaluate the overall 3D performance of an instrument. They rely on indoor or outdoor testing facilities with known and well-distributed marker fields or well-defined 3D objects in the 3D scene. However, the issue with these system investigations is that single processes are often hard to be separated from others in the rather complex processing chain of TLS data. To obtain more detailed knowledge about a specific process (like the distance measuring performance), all other processes have to be isolated and thus kept constant in a specific experimental setup.

In this study, the precise investigation of the distance measuring performance was performed using a sophisticated linear comparator system with an independent, highly accurate and stable reference measuring system.

**Figure 3:**
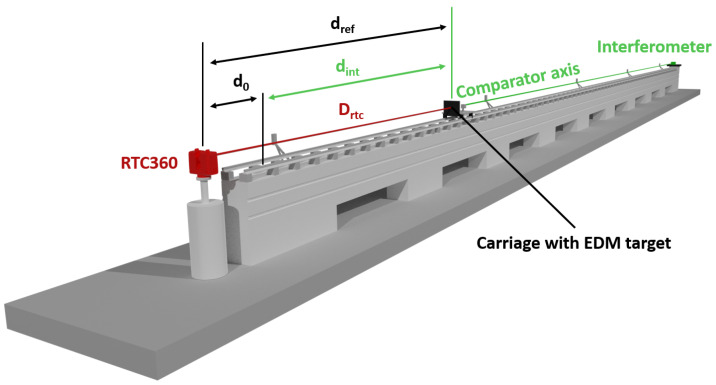
Schematic setup of the RTC360 on the 30 m long horizontal comparator.

In Figure 3, a schematic setup of our RTC360 tests is depicted. The RTC360 carries out a panorama scan and uses it to measure the distances $D_{rtc}$ to an EDM target plate on a remotely controlled carriage. The initial distance to the EDM target, relative to the scanning center, $d_{0}$, is determined with a calibrated total station (Section 4.3). The carriage’s position along the comparator bench $d_{int}$ is measured using an interferometer (Section 4.3). Both distances together provide the “true” distance $d_{ref}$, which will be compared to the RTC360 distance $D_{rtc}$. The distance deviation $\Delta_{d}$ is computed as follows:

(1) $$\Delta_{d}=\left(d_{0}+d_{int}\right)-D_{rtc}$$

Contrarily to the testing of total stations or handheld “Distos”, where the distance between the device and the moveable target can be directly (and thus quickly) measured, deriving a representative and comparable $D_{rtc}$ out of each panorama scan from all $D{\left(i\right)}_{rtc}$ is more ambitious. Due to the predefined scanner resolution and the unknown internal sampling, it is, in general, not possible to measure a single EDM point directly on the comparator axis. Also, selecting the most representative single EDM measurement (e.g., with a nearest neighbor search), is not supposed to be suitable due to the assumed lower scanner resolution at the end of the comparator bench.

Thus, we decided to use a set of single EDM measurements $D{\left(i\right)}_{rtc}$, close to the comparator axis, to derive an “averaged” distance $D_{rtc}$, which will be used further on. All single $D{\left(i\right)}_{rtc}$ within a 15 × 15 cm^2^ large section are used to fit a plane representing the flat surface of the EDM target (Section 5). $D_{rtc}$ is derived as the distance from the scanner center to the intersection of the estimated plane with the comparator axis. The possibility of accurate plane fitting is supported by the high reproducibility of the single EDM measurements given in the instrument’s data sheet [3]. Furthermore, this strategy tributes the manufacturer’s approach during the calibration of the scanner, where flat targets are used at two different distances. Also, in many practical applications of laser scanning, a subset in the point cloud (e.g., representing a wall or a floor) is usually more important than a sole data point.

During the experiment, a vast number of scans were performed of the EDM target at different positions, and all these scans form a time series of the data. In order to exclude possible effects of the co-registration of scans on the results, the RTC360 was physically fixed on a stable surveying pillar during the experiments. The internal coordinate system provided the relation between each scan, and any shifts in the internal coordinate system were avoided by switching off all auxiliary positioning processes (e.g., IMU, tilt, VIS…). The stability of the internal coordinate system, using the API of the RTC360 to extract and convert the raw data (because only a minimum of processes are applied to the raw-data), was indicated in a prior study [17]. Nevertheless, additional stable scanning targets were placed in the scene throughout the experiments to additionally monitor the stability of the set-ups during the 24 h experiments.

The comparator investigations were repeated with a set of EDM targets (see Section 4.2) to obtain representative results for high-reflective surfaces, medium-reflective surfaces and low-reflective surfaces.

## 4. Laboratory Set-Up

### 4.1. Geodetic Metrology Laboratory and Its Horizontal Comparator

**Figure 4:**
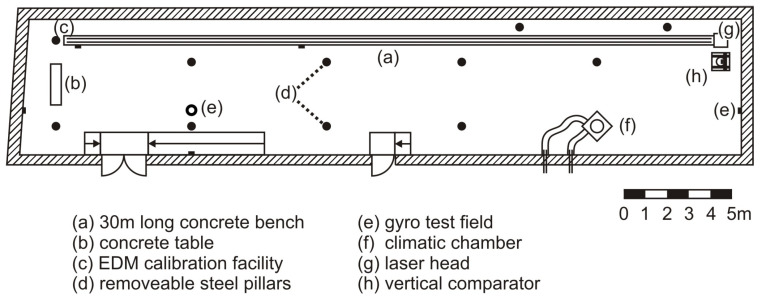
Schematic layout of the geodetic metrology laboratory at TU Graz.

Figure 4 shows a schematic layout of the geodetic metrology laboratory (GML) at the Graz University of Technology. It is located on the ground floor of a university building with a foundation that is separated from the foundation of the building. This is an important fact, as the foundation (and everything connected to it) is not influenced by temperature-, wind-, or traffic-induced movements of the building. The laboratory has a size of 33.2 × 6.3 × 3.5 m^3^, has no windows (no daylight), and is climate-controlled (temperature: 20.0 ± 0.5 °C, humidity: 50 ± 10% r.H.), and thus, it provides the stable conditions that are needed for instrument testing.

One of the main facilities in the GML is the EDM calibration facility that consists of a 30 m long concrete bench (a), equipped with a rail system, where a carriage can be fully automatically moved within an EDM distance range from about 0.8 m to 29.6 m. The concrete bench is slightly inclined (1.6 mm/30 m), and the rail system is linear with vertical deviations of less than 0.4 mm. On one side of the carriage, a corner-cube reflector is mounted for the reference measurements, which are performed using a laser interferometer. Five temperature sensors (located along the bench), one air pressure sensor, one humidity sensor, and one carbon dioxide sensor are used for the meteorological reduction in the interferometric measurements. A central computer controls the whole system and initiates the measurements. The positioning accuracy of the carriage along the horizontal comparator is about 0.02 mm due to mechanical reasons. However the positions are precisely measured using the interferometer. The maximum stage stability during the time of a RTC360 panorama scan (about 1 min, including the time for data storage) is about 0.005 mm. The expanded standard uncertainty (*k* = 2) of the horizontal comparator measurements $d_{int}$ was estimated according the ISO/IEC GUIDE 98-3 “Guide to the Expression of Uncertainty in Measurement” (GUM) [18]; see Equation (2). Thus, the maximum standard uncertainty for the comparator measurements (distance < 30 m) is about 0.01 mm.

(2) $$Ud_{int}=\pm \sqrt{{\left(3.4\;\mu m\right)}^{2}+\left(0.29\cdot 10^{-6}\cdot L\right)};\left(k=2\right)$$

The laser scanner was mounted on a stable pillar (c) and initially aligned to the height of the interferometer’s laser beam in order to reduce Abbé errors, using a total station at the scanner’s position.

The EDM targets were attached to a flat (CNC manufactured) aluminum plate of 32 × 27 cm^2^ in size and 20 mm in thickness. At the back side, this target plate was equipped with an adjustable bearing device that allows the alignment of the plate orthogonal to the comparator axis. The alignment of the EDM target plate was also performed with this total station using an auto-collimation mirror at the target plate.

Aside from the comparator, several scanning targets were placed in the laboratory on different stable steel pillars in order to reveal possible rest drifts in the scanner measurements and/or its internal coordinate system.

### 4.2. Selection of EDM Targets

For highly frequent reflectorless measurements, no standards are available yet. Therefore, the ISO 16331-1:2017(E) [19], which specifies the testing procedure of handheld EDM instruments, was considered the closest related standard. The ISO 16331-1:2017(E) involves matt white self-adhesive foils (like those from 3M or Avery) that are attached to a glass or aluminum plate as an example for suitable targets. Further, the use of targets with a specified reflectance (like Spectralon^®^ material) are proposed for the relative determination of the reflectance of the chosen targets. However the Spectralon^®^ targets should not be used for distance measurements because the laser beam penetrates the material up to a few millimeters and the penetration depth also changes with distance. As the Avery 501 matt white foil has already been already successfully used with the horizontal comparator for testing handheld EDM instruments, it ought to be a good candidate for the scanner tests representing high reflective targets.

Furthermore, a low-reflective target should be used, especially for the RTC360, because of its low and a high energy pulse. The manufacturer specifies accuracy information in the instrument’s manual for three tested reflectivities; see Table 1. This provides some guidance for the target selection, but it was not stated in [12] which specific type of target was used within the manufacturer tests.

**Figure 5:**
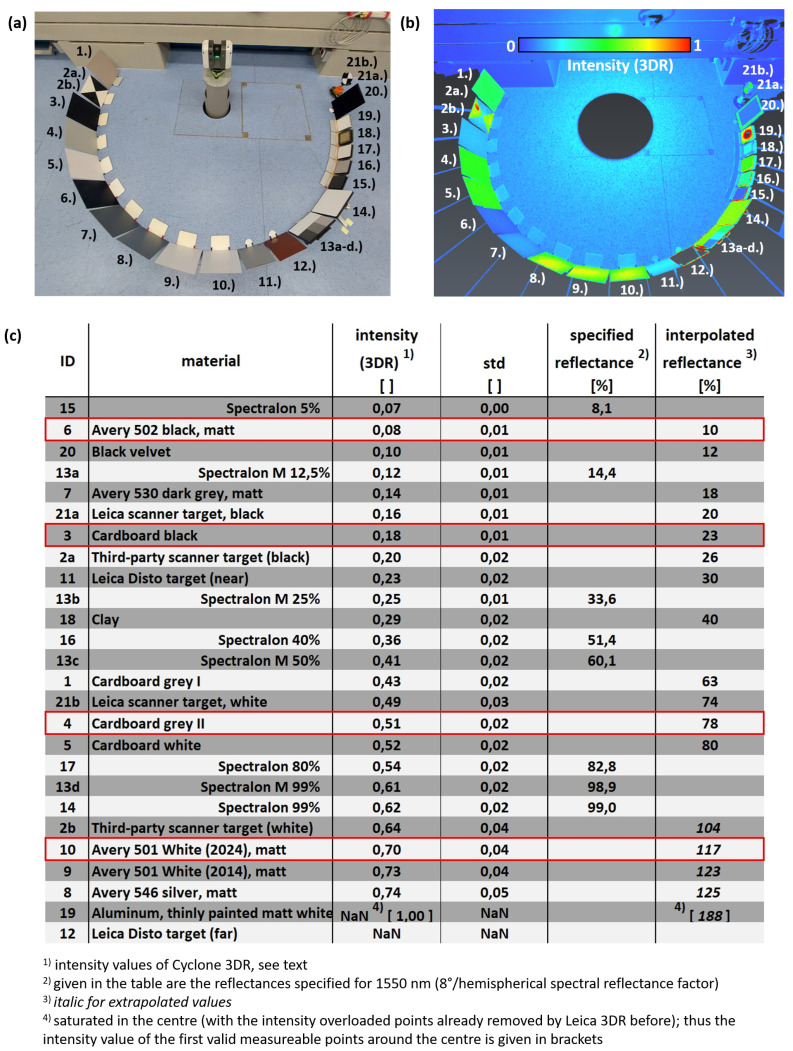
(**a**) Setup for the determination of the reflectance of a variety of different targets, (**b**) intensity-colored point cloud, (**c**) intensities of different targets, measured with the RTC360, and their reflectance derived from the Spectralon^®^ targets; targets chosen for the comparator investigations are in red.

In order to find other suitable targets with an adequate reflectance, a selection of different surfaces (flat, 27 × 32 cm^2^ large cardboards and other Avery films, scanning targets) was set up under the same conditions (distance from the scanner of 1.48 m, orthogonal alignment to the scanner center using an auto-collimation mirror); see Figure 5a. These targets were scanned with the RTC360 together with different Spectralon^®^ diffuse reflectance targets (10″ and 5″).

For the purpose of the intensity determination, the processing of this scan had to be carried out with the manufacturer workflows of Cyclone Register 360 and Cylone 3DR (Leica Geosystems). The export with the API does not provide detailed intensity information. Therefore, all intensity information in this paper relates to numerical values provided by Cyclone 3DR after the standard filtering in Cyclone Register 360, with numerical values within the range of 0 and 1. Figure 5b shows the intensity-colored point cloud of the test setup. At some targets, the intensity varies over its area, with maximum values close to the center. Thus, numerical values for the comparison were derived in this maximum central region for further use. These are listed in Figure 5c, together with their standard deviations and interpolated values (using the specifications of the Spectralon^®^ targets).

The intensity values show that Avery 501 white (10) is far away from saturating the photodiode (Register360) removes the points with intensities > 1.0 automatically if the corresponding filter option is chosen), and it was thus used in the experiment as an example of a high-reflective target. Avery 502 black (6) is the target with the lowest reflectance within this test, even a little lower than the black velvet (20). Thus, it was also chosen for the experiments.

The Leica scanner target GZT21 (21) would also be a candidate for the investigation, as it is part of the scanning system, and thus, one might assume that it was optimized for this. However, it is too small (diameter of 11.5 cm) for the comparator measurements, as at the 30 m distance, very few undisturbed points might appear at each target section. As an alternative, the bigger third-party scanner target (2) was considered; however, the reflectance of the white section significantly differs from the one of the Leica scanner target. An interesting detail for this target can also be seen in Figure 5b, with a highly reflective region in the white section. There, this used target was cleaned using isopropanol to remove some dirt, which obviously destroyed the cover layer of this target and resulted in an increased reflectance.

Anyway, the black (3) as well as the gray photo-cardboard (4) show a comparable reflectance to the two sections of the Leica scanner target and are also close to the Leica disto target (11). As cardboard is also used by some manufacturers to test their laser scanners [14], the black and the gray cardboard were also included in the investigations. Further, this choice of targets is close to the the reflectivities used by Leica for their specifications; see Table 1.

### 4.3. Determination of the Initial Absolute Distance $d_{0}$ from the Scanner’s Center to the Targets

As the interferometer delivers only relative measurements $d_{int}$ along the comparator, further measurements were carried out to determine the initial comparator distance $d_{0}$ between the scanner and the EDM target plate. This should provide a rough absolute reference distance. However, as the measuring center of the scanner is not physically accessible, $d_{0}$ cannot be determined as precisely as the interferometer distances. Therefore, the absolute distance deviations will show a lower level of accuracy compared to the relative distance deviations.

A calibrated Leica TS15 total station was used for the determination of $d_{0}$ at an approximate distance of about 0.77 m from the scanner center. It was mounted on an appropriately located steel pillar in the laboratory to realize a line of sight almost orthogonal to the comparator bench. Then, $d_{0}$ is mostly determined using the more precise angle measurements of the total station instead of its less accurate distance measurement. Assuming that the Leica’s centering concept is also valid with the RTC360 (the adapter for the RTC360 is manufactured and distributed by Leica), the position of the scanner was realized using precise geodetic forced centering equipment.

**Figure 6:**
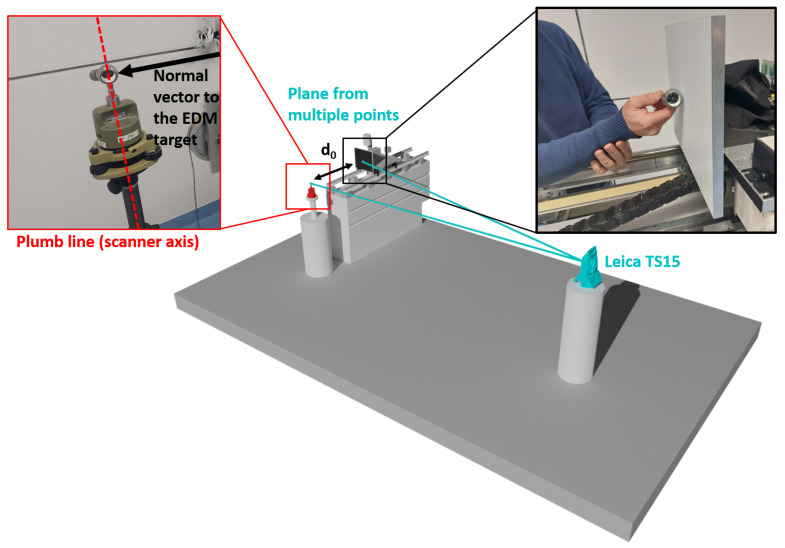
Schematic setup for the determination of $d_{0}$ with the used Leica TS15 orthogonal to the comparator bench.

This implies that the scanner has to be set up vertically, which was realized by adjusting the tribrach that is attached to the instrument-holding pillar of the horizontal comparator. Here, the electronic compensator of the TS15 was used. The frontside of the EDM target was measured with a sphere-mounted retro-reflector with a 38.00 mm diameter and an eccentricity of about 0.1 mm; see Figure 6. On the EDM target plate, several points were measured to estimate a plane and compute the orthogonal distance ($d_{0}$) between this EDM target plane and the horizontal scanner center.

It can be seen in Table 2 that the standard deviation for each estimated $d_{0}$ is less than 0.1 mm. In taking some minor centering errors of the reinserted scanner into account, as well as the eccentricity of sphere prism, the absolute distance $d_{0}$ should be better than 0.3 mm for all EDM targets. The variation in $d_{0}$ comes mainly from the thicknesses of the targets.

## 5. Automation of the Test Set-Up, Point Cloud Processing, and Estimation of $D_{rtc}$

The usage of the scanner API enabled a high degree of automation of the laboratory experiments. A laptop with Matlab was used to coordinate the scanner measurements (triggered with the API) and to communicate with the horizontal comparator. Although the comparator measurements did not require any further human interaction, the measurement and computation times, as well as the data storage of the RTC360 data, were critical issues after all.

During the experiments on the comparator, the raw data were stored on the mandatory 200 GB internal USB stick in a project.rtc360 file structure. A limiting factor was the conversion time of the project.rtc360 files into further .csv files. This can take a considerable amount of time for each single scan with the API and scales up with the scanner resolution and file size.

Thus, in order to be able to execute the experiments and postprocessing within a reasonable amount of time, a compromise between scan resolution and comparator step width had to be found.

Therefore, we decided to carry out the experiments with a 4 cm step width on the comparator both ways, with a 2 cm shift for some selected experiments between the forward and the backward run. To keep the point cloud data and conversion time low, the scans were taken with a resolution of 12 mm @ 10 m. This choice does not limit the interpretation of the gathered data, as the manufacturer states no difference in accuracy between the scan resolution modes [12].

Each EDM target investigation resulted in 1450 panorama scans, with a raw-data file size of approximately 50 GB and an overall measurement time of 24 h. The conversion of the project.rtc360 files with the API into .csv files took 4 days for each measurement series, using standard hardware (i.e., the steering laptop).

The .csv files were further processed on a performant personal computer with a Python script, using the open3D toolbox [20]. The section of the point cloud that contains the EDM target was roughly clipped with a cubic bounding box (0.5 × 0.5 × 0.5 m^3^). In order to automatically detect the flat EDM target inside this bounding box, a RANSAC algorithm (3000 iterations, distance threshold = 0.005 m) was used. As the selected points may still contain mixed distances at the edge of the EDM target, due to the RANSAC threshold, only a central 15 × 15 cm^2^ area was used for the final plane estimation afterward. Using this, a safety zone of at least 45 mm to the edges of the target was established.

All individual distances $D{\left(i\right)}_{rtc}$ within this 15 × 15 cm^2^ area contribute to the representative scanner distance $D_{rtc}$, which is computed as the distance from the scanner to the intersection point of this final plane estimation with the comparator axis.

**Figure 7:**
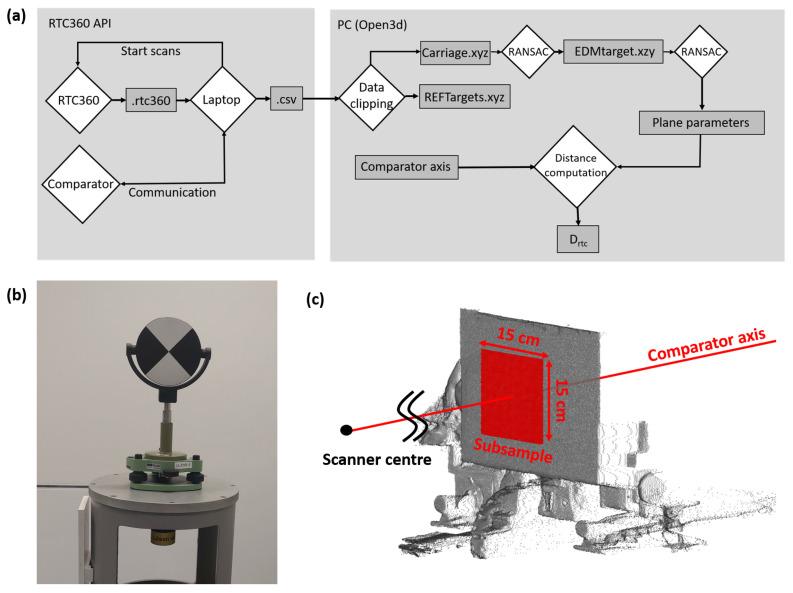
(**a**) Processing workflow of the point cloud data, (**b**) Leica GZT21 scanner target for stability control, and (**c**) clipped comparator EDM target with carriage and final plane estimation.

After processing, the distance $D_{rtc}$, the standard deviation $sD_{rtc}$, and the number of contributing points are exported into an ASCII file for further visualizations. The whole workflow and the plane detection are schematically visualized in Figure 7.

The clipped carriage point cloud and the EDM target point cloud are exported with a Python script in the .xyz format and provided in the TU Graz repository to the community [11]. The clipped reference target point cloud (an exemplary target is seen in Figure 7b) is stored separately for documentation purposes.

## 6. EDM Target #1: Avery 501 White

### 6.1. Performance along the Whole Comparator Distance

**Figure 8:**
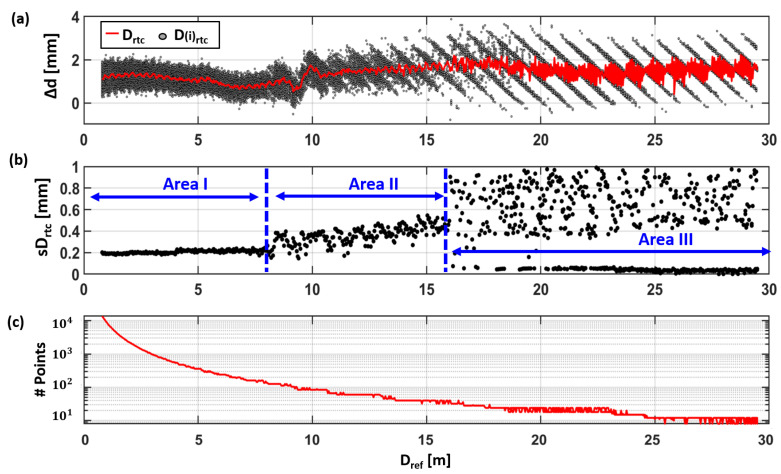
(**a**) Distance deviation of the RTC360 measurments to the reference distance, (**b**) standard deviation of the distance residuals, and (**c**) the number of contributing points to the plane estimation.

On the horizontal comparator, low-resolution (12 mm @ 10 m) scans on Avery 501 white foil were carried out with a spacing of 40 mm along the comparator bench. Figure 8a shows the distance deviations of all individual scanner measurements ($D{\left(i\right)}_{rtc}$) (within the 15 × 15 cm^2^ subsample) as gray points. The deviations $\Delta_{d}$ (Equation (2)) of the scanner distance ($D_{rtc}$) to the reference distance are shown as a red line. In Figure 8b, the standard deviation of the distance residuals of the plane estimation can be seen. Figure 8c shows the corresponding number of contributing points to the plane estimation.

When focusing on the single measurements (gray points) different systematic behaviors become evident. Figure 8a reveals an absolute distance offset for the used TU Graz RTC360 (Firmware: 6.01.131) of approximately 1.3 mm and an isolated pattern at a distance of about 10 m with distance deviations of about 1.2 mm within a distance range of 1.6 m (see red line). Thus, for further interpretation, the data set was divided into three areas according to the different stochastic properties (see Figure 8b). Despite the aforementioned distance offset, the deviations $\Delta_{d}$ of all distances $D_{rtc}$ are within a range of about one millimeter in Area I. The single scanner points $D{\left(i\right)}_{rtc}$ show stochastic behavior on the EDM target, with a standard deviation of 0.2 mm, which is slightly increasing up to a distance of approx. 8 m. In having a more detailed look at the more precise “averaged” distances $D_{rtc}$, a cyclic-like pattern with a period of approx. 30 cm and a very small amplitude of about 0.1 mm to 0.2 mm can be seen in Figure 8a. It is also slightly increasing with the distance, but still much lower than the specifications of the scanner. Also, a long periodic wave of a very small amplitude might be overlaid.

In Area II, between 8 m and 16 m, larger standard deviations are shown (see Figure 8b) as well as an increase with distance. However, there are also some—maybe periodic—sections of smaller noise.

Then, starting at approx. 16 m up to the end (Area III), the behavior changes completely, with an initial transition zone of some meters. At some distances, all points at the EDM target fit well together, resulting in a very small standard deviation of <0.1 mm, but at other positions, it increases up to 1 mm, which was investigated later in some more detail.

However, it has to be kept in mind that with larger distances, the number of reliable measuring points within the final target plane becomes less. Figure 7c shows this number of measuring points on the target, which decreases from more than 13,000 points down to 36 points (at 20 m) and to only 8 points (at 29.5 m), for the chosen scanner resolution (12 mm @ 10 m); see Figure 8c.

Here, one would ask to use the 3 mm scanner resolution. However, keep in mind that this would come with a much longer processing time in the workflow. This was the compromise taken to see possible short periodic errors expected by the scannner’s WFD within a reasonable processing time.

Anyway, it can be summarized that the distance deviations in Figure 8 show a systematic behavior, which will be investigated in the next section in more detail for Avery white.

### 6.2. Performance at Selected Distances

Four distances showing different behaviors were selected. For these, the intensity values were also included in the evaluation. The four distances are as follows:

- 0.8 m, starting position in Area I with a maximum number of points and the highest variation in incidence angles.
- 10.0 m, position in Area II; here, the range noise is specified by the manufacturer [3].
- 19.8 m, position in Area III; the standard deviation in the measurement series is very low.
- 20.0 m, position in Area III; close to 19.8, but the standard deviation is high and close to 1 mm. Furthermore, this distance is also used in the manufacturer’s noise specification [3].

**Figure 9:**
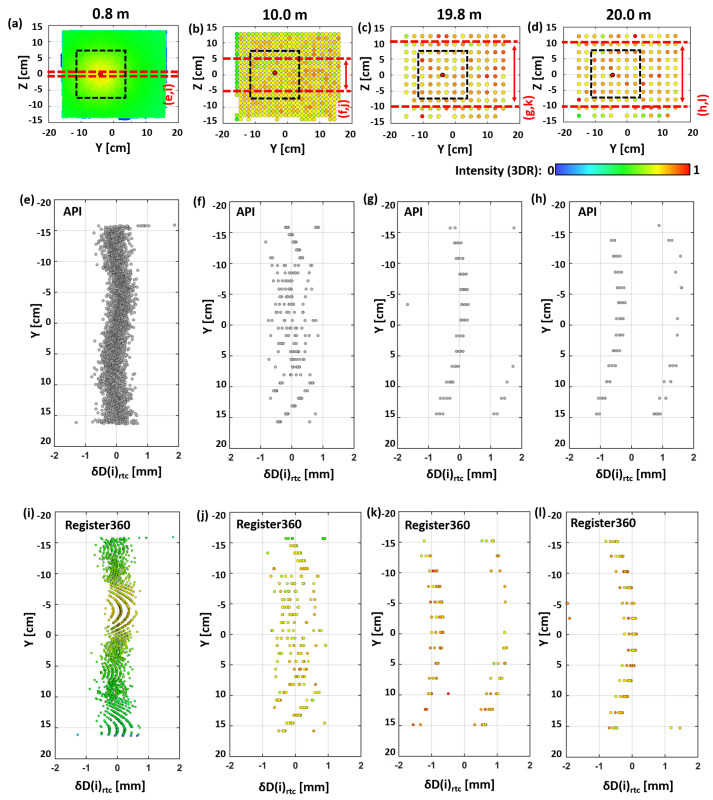
(**a**–**d**) Intensity-color-coded front view of the whole EDM target plate at four distances of different behaviors, (**e**–**h**) corresponding distance deviations of the API-processed point clouds (ground view), and (**i**–**l**) the intensity-color-coded distance deviations of the same Register360-processed point clouds.

In Figure 9, different point cloud data for the four selected distances are shown. Figure 9a–d, show the intensity-coloured point cloud at the whole EDM target. It has to be noted that for all intensity plots, Leica Cyclone Register and Cyclone 3DR had to be used again. In the subplots, the position of the central 15 × 15 cm^2^ plane is also indicated by the dashed box in order to also obtain a visual impression of the intensity variations inside the subset and the decreasing point density with distance. The red and dashed lines show the data sections used for the other subplots below, where the distance deviations are plotted in the ground view for the shown point clouds. Further, it can be seen that the EDM target was mounted 4 cm eccentrically in the Y axis, compared to the comparator axis, due to mechanical reasons.

For subplots (e–h), the point clouds were processed as before, using the API, and thus these are the same data shown in Figure 8. In order to color the distance deviations $\delta D{\left(i\right)}_{rtc}$ by the measured intensity values in Figure 9i–l, these point clouds were “manually” converted and processed with the manufacturer software using the standard filters.

In Figure 9a, the central area with nearly orthogonal incidence angles can be distinguished from the rest of the EDM target plate by its higher intensity values. The distances within the horizontal cut (±1 cm in height) in Figure 9e show measurement noise that is not significantly systematic, which one might assume due to the varying angle of incidence and intensity values. As previously mentioned, the API-processed distance deviations could not be color-coded as there was no detailed intensity information provided.

Surprisingly, in Figure 9i (with a further data cut for clarity: ±3 mm in height), with the newly processed point cloud (Register360), strange systematic patterns appear with a clear deterministic behavior and a correlation with the intensity values in the central area.

However, this pattern seems to be copied over the target plate every 10 cm, with no dependency on intensity any more. This effect might be an artefact of the internal filter process in the software, which cannot be turned off by the user in Register360. This ought to be interesting for the many users of this standard software, especially when a data evaluation is carried out within the millimeter range or a correlation between the stochastic behavior and intensity value is further investigated.

At 10 m, both Figure 9f,j show the same overall behavior, with a stochastic pattern in the central area and a systematically striped behavior at both outer sides. A pattern like this will be seen more clearly in Section 8 as a GZT21 scanning target but for a shorter distance. Anyway, the intensity values at 10 m are not correlated with the pattern and seem to be rather randomly distributed within the shown point cloud. Also, the previously observed “10 cm” copies of the pattern visible in the shorter distance (Figure 9i) do not appear in Figure 9j, maybe because the target size is too small to see here.

For the distances of 19.8 m and 20 m, the central stochastic pattern is gone, and the striped pattern fully dominates, with a different number of points in each stripe. Changing from the API (Figure 9g) to the standard workflow again (Figure 9k) does not change the pattern, but obviously, some further corrections are applied. The curved stripes are separated by about 2 mm from each other. This results in a low standard deviation (<0.1 mm) for the data in Figure 9g (almost all data are in one stripe) and a standard deviation close to 1 mm for the data in Figure 9h (here, the points are more equally distributed in both stripes). The corresponding intensities are almost identical at both distances.

Anyway, the reason for these stripes and their distinct curved appearances remains unknown, and even investigating their relationship with the intensities does not provide more information. The structure is most likely linked to the distance resolution capabilities of the WFD and the flat surface. The reader might obtain a more detailed impression about this behavior by viewing the evolution of this pattern over all distances (for the API data) in the provided videos in our repository [11].

**Figure 10:**
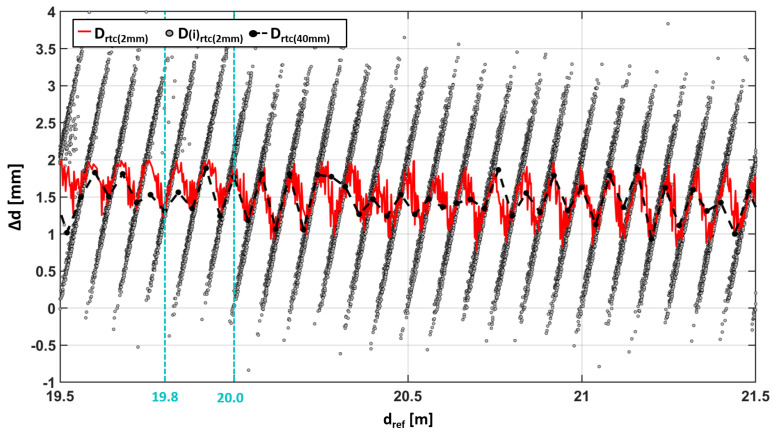
Detailed 2 mm step width investigation of Avery white between 19.5 m and 21.5 m.

**Figure 11:**
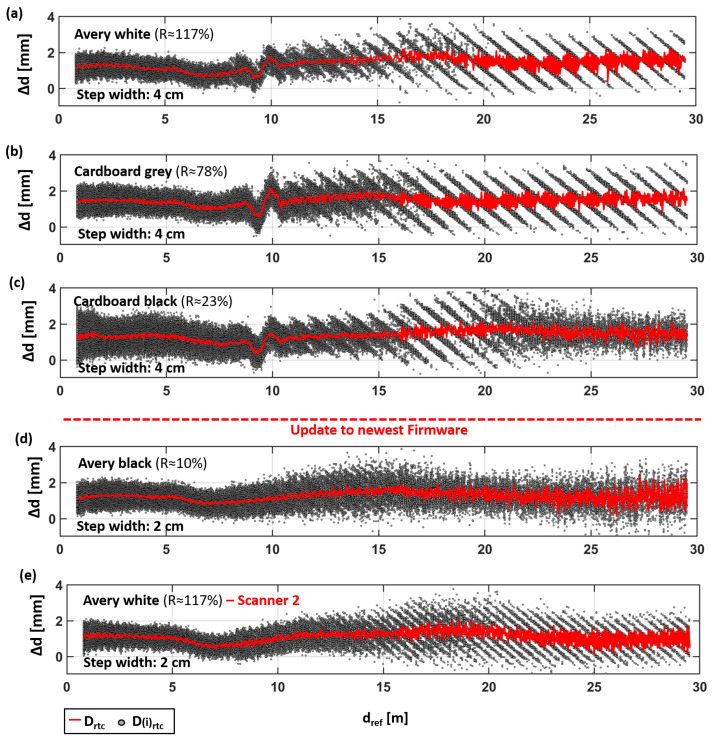
Distance deviations at (**a**) Avery white, (**b**) cardboard gray, (**c**) cardboard black, (**d**) Avery black, and (**e**) a repetition of Avery white with a second scanner (also showing the reflectance R).

However, it can be said that the relative distribution of the different single points in the patterns result either in the low or high standard deviation that were shown in Figure 8b. This system was studied in more detail in Figure 10 with a measurement series for Avery white, which was recorded with a 2 mm step width on the horizontal comparator in a range from 19.5 m up to 22.5 m. It can seen that the number of “cycles” of $D_{rtc}$ is increased by the higher resolution on the comparator, compared to the measurement series with a 4 cm resolution (Figure 8a). This shows that the number and appearance of the cycles shown in Figure 8a are affected by an aliasing effect. This is depicted in Figure 11, where the corresponding data of Figure 8a are overlaid as black and dashed lines on the 2 mm data. Although, they originate from different experiments, they fit well to each other and demonstrate the reproducibility of the effect.

Anyway, dedicated experiments with much smaller distance steps are planned for the future in order to focus on the distance resolution capabilities. The inclined lines in Figure 11, which arise from the $D{\left(i\right)}_{rtc}$ at different positions along the comparator, are also conspicuous. In order to link the striped pattern to Figure 9g,h, two vertical lines are drawn in Figure 11 at the same positions. These intersect with the inclined lines at the two positions of the two curved stripes.

Using the information gathered up to now, it becomes obvious that the point distribution between each stripe shows a clear correlation with the measurement distance and repeats itself. This effect causes the conspicuous behavior of $D_{rtc}$ in Area III.

## 7. Comparison of Four Different EDM Targets

In Figure 11, the result gathered with the other EDM targets are shown in comparison to the data of Avery white (Figure 11a is identical to Figure 8b and is shown as a better visual interpretation for the reader).

**Figure 12:**
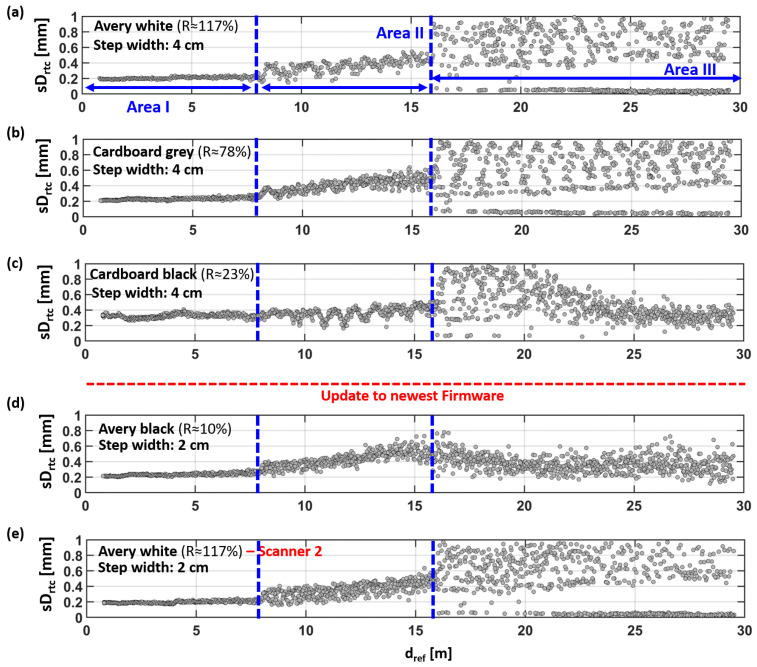
Standard deviations of the distances $D_{rtc}$ at (**a**) Avery white, (**b**) cardboard gray, (**c**) cardboard black, (**d**) Avery black, and (**e**) a repetition of Avery white with a second scanner (also showing the reflectance R).

It can be seen that a distance offset is inherent with all EDM types and that there are also areas of different behaviors. These areas are quite the same for cardboard gray. Contrarily, for cardboard black, in Figure 11c, it can be seen that the deterministic pattern in Area III changes again to a more stochastic behavior at 21 m. In Figure 11d, the deterministic pattern is significantly reduced with Avery black and barely visible any more; see also the corresponding standard deviation in Figure 12d.

However, for all reflectance values, the absolute distance deviations to the estimated planes (red lines) remain below 2 mm for the tested distances up to 30 m.

The deviations of all single points, within the 15 × 15 cm^2^ subsets, show absolute deviations of less than 4 mm, although differences in the behavior can be observed between the materials. It is shown in Figure 12 that Avery white and cardboard gray show a similar behavior, except for a slightly increased standard deviation in Area II for cardboard gray. Cardboard black shows the highest standard deviation in Area I, which is close to the specifications of the instrument. Up to 20 m, it is similar to the other two, but from there on, the deviations become more stochastic again.

Avery black demonstrates a more homogeneous behavior in Figure 12d, but also, it is the most different to the other targets. All these data indicate that different measuring modes are used by the scanner and that the transition between these modes is dynamic, partly overlapping, and somehow related to the reflectance and distance. However, for a better understanding of the individual processes, detailed knowledge about the internal functions and corrections of the scanner would be needed, which are not shared by the manufacturer to the general public. Furthermore, the behavior of the scanner might also change over time (e.g., due to a firmware update).

This can be seen in the aforementioned isolated pattern (at about 10 m), which is observed in Figure 11a–c, but is with the Avery black target in Figure 11d. Although a correlation with the material change would be obvious, here, this change is more likely related to a forced firmware update (upgrade from 6.01.131 to 6.03.191) before the measurements of Avery black were carried out. Afterward, the TU Graz scanner was unavailable for further investigations due to repair work. Thus, for Avery white, the measurements were repeated with a second RTC360 scanner also on the newest firmware level (6.03.191), as shown in Figure 11e. The results are almost the same as those seen for the TU Graz scanner (with the lower firmware level) in Figure 11e. However, except for the artefact at 10 m, which is also missing with the same firmware level now, it can be assumed that this artefact (in the millimeter range) was removed with the firmware update. This is an issue that has to be taken into account for precise tasks (e.g., monitoring applications).

## 8. Point Cloud Behavior at a Selected Leica Reference Target (API Data)

A side effect of several hundred scans, which were carried out in each comparator measurement series, is that the surrounding objects in the laboratory have also been scanned several hundreds of times. With this huge amount of captured data (all under the same conditions), the stochastic behavior of the scans becomes more visible and provides some detailed insights into the working principle of the device.

**Figure 13:**
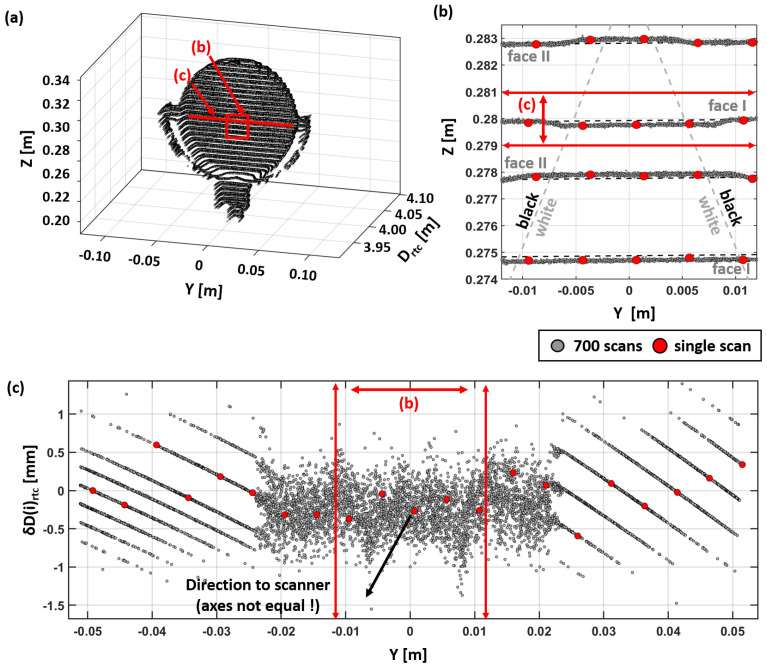
(**a**) Aggregation of several hundreds of single scans on a reference target GZT21, (**b**) zoomed front of the target, and (**c**) ground view of a single “horizontal measurement line” on the target.

The data shown in Figure 13 consist of about 700 scans as a part of a single comparator experiment. The original intention of using scanner targets was to prove the stability of the scanner’s coordinate system during each experiment. One of these Leica GZT21 targets is shown in Figure 13, which was placed at a 4 m distance to the scanner and was only roughly (manually) aligned to the scanner. This did not only prove the stability throughout the experiments (no detectable shifts), but also, the systematic behavior of the points on the target’s surface became visible, which will be further examined in this section.

Figure 13a shows a 3D view of the target with the overlaid 700 scans. A distinct pattern with horizontal lines became visible. The vertical spacing reveals the fact that the scanner measurements are triggered by the rotating mirror and, thus, are almost at the same vertical angle in each scan. The observed spacing of approximately 5 mm at a distance of 4 m corresponds well to the chosen measurement mode (12 mm @ 10 m). Contrarily, the horizontal spacings show a more stochastic pattern with the points being much closer to each other, forming visible horizontal lines. This indicates that the vertical scanning lines of individual scans are not directly reproducible. The scanner’s horizontal starting point slightly changes from set-up to set-up. However, horizontal angle readings of the RTC360 were carried out (like in a total station) using angle encoders. The orientation of the internal coordinate system was fixed by its graduated circle, and thus, the geometric relation between the measured points of different scans was preserved. The variation in the vertical scanning lines is a direct result of the limited positioning accuracy of the scanner.

In Figure 13b, a smaller section of the target’s front face (close to its center) is shown. Additionally, the crossover from the black to the white section of the GZT21 target, and vice versa, is depicted by the dashed gray lines. Four measurement lines are depicted in Figure 13b within a 10 mm height interval; this is double of that of the expected two lines with the selected 12 mm resolution at 10 m. This occurs because the RTC360 scans horizontally at slightly more than 180°, and thus, there is a small overlap between the face I and face II measurements. In the experiments, the scanner was roughly aligned orthogonal to the comparator bench at the beginning to avoid this “zero-point transition” at the comparator’s EDM target. However, this is why the overlapping zone was then on the left-hand part of the shown target, as the holding pillar is also orthogonal to the comparator axis.

In Figure 13b, at the border region, between the black and the white sections of the target, a small variation in each horizontal measurement line can be seen (elongated with dashed lines for better visibility): once above the line, and the next one below. This little offset is caused by the 75 ns temporal offset between the low- and high-intensity pulse of the scanner, which are used for the distance measurement. The first pulse is used for measurements in the dark section, and the second pulse, for the bright target section. This results in a short delay in one or the other direction, depending on the direction of the mirror rotation of the two faces in the overlap.

Figure 13c shows more comparator measurements. It shows the distance variations along the reference target in a small vertical section, about 5 mm below the target’s center. In the inner section of the target (about ±22 mm), the distances show rather stochastic behavior, with some exceptions like the vertical patterns at −6 mm and +8 mm, which correspond again to the border region between the black and the white sections of the target. More surprising is the behavior outside this central region, which shows strong systematic patterns with obviously discrete distance steps. These steps change over the target and also vary in their inclination, most likely because the target was not perfectly aligned to the scanner (the axes in Figure 13b are not equal). However, they show a behavior similar to that of the striped pattern (previously seen in Figure 9) in Area III.

Anyway, this example shows that the different distance measuring modes (or corrections) are chosen based on the scanner, whilst the measurement and may not be seen in a single scan. Here, the sampling is usually too low, or it is smudged by other effects (e.g., registration), which misleads the user to assume a rather random distribution in the measurements.

## 9. Summary and Discussion

The horizontal comparator in the geodetic metrology laboratory at the Graz University of technology was used to investigate the distance performance of the RTC360 laser scanner on different target materials (see Section 6.1 and Section 7) in a distance range from 0.8 m to 29.5 m with 2 cm and 4 cm steps. The investigations showed some systematic behaviors in the measurement series with magnitudes of a few millimeters. The observed distance offset, of about 1.3 mm, would make a scanned room about 2.6 mm smaller between two opposing walls. A comparable distance offset was also observed with a second RTC360.

Furthermore, systematic deviations at a 10 m distance (for the older firmware version) and other pseudo-cyclic effects were found. Distance deviations with stochastic behavior appeared, as well as deviations with distinct deterministic behavior. Targets with higher reflectance (e.g., Avery white and cardboard gray) showed this distinct measurement behavior and an incoherent standard deviation beyond a distance of 16 m in the given setup. Contrarily, a target of lower reflectance (cardboard black) showed that after a distance of 21 m, the behavior can become stochastic again. For the lowest reflectance (Avery black), the deterministic parts had nearly vanished. The operation modes of the RTC360 for distance measurements seem to be dependent on the material and the distance. Surprisingly, the relative distribution of the intensity values on the EDM target did not noticeably affect the measurement results. Also, it was demonstrated again that firmware updates can have significant influence on the distance measurement performance (the presumable removal of the 10 m artefact).

Although several systematic behaviors were revealed in the scanner distances $D_{rtc}$, the RTC360 fulfills the manufacturers’ specifications in terms of the given 3D point accuracy (see Table 1). The specification of about 6 mm at 20 m (95%) was not exceeded by any of the taken measurements. Anyway, the specified “Range noise**” and *“For single shot measurements”* [3] specifications should be critically discussed and clarified by the manufacturer for the current firmware version. In Section 6.2, it was discussed that the distance deviations of the RTC360 are neither random nor normally distributed. The deterministic striped pattern (presumably due to the sampling resolution of the WFD) demonstrates that the distances have discontinuities up to 2 mm at 20 m. The relative single-point distribution between two adjacent stripes results in a pseudo-cyclic behavior of $D_{rtc}$. The more detailed investigations for Avery white (with a step width of 2 mm) showed that this pattern might be better described by a saw-tooth pattern.

These finding have to be taken into account when carrying out tasks that require a high relative accuracy between neighboring points (e.g., fine-structured objects). Due to the deterministic parts in the distance measurements, obtaining a reliable object roughness with RTC360 scans that are better than these two millimeters seems to be unlikely without further filtering. Furthermore, the different processing approaches of the data with the API and Register360 demonstrated once again that the results of laser scanners are highly related to the applied workflow. Intensity-based systematics in the RTC360 point cloud (processed with Cyclone Register 360) turned out to be artefacts of the internal software filters and are not present in the raw data, which were directly extracted with the scanner’s API. This demonstrates that significant filtering is already applied by the manufacturer’s processing software.

Overall, the experiments presented in this paper provide an overview of some systematic effects that are linked to the distance measurement of the RTC360 and confirmed some details about the measuring process. The proper interpretation of the effects is challenging, as there is little knowledge available about the internal processes (WFD). However, the presented results should support further research and provide more realistic accuracy expectations for field surveys with the RTC360. To allow further investigation beyond the scope of this study and to support a wider discussion in the community, complementary research material is provided in the TU Graz repository for download under the Creative Commons Attribution 4.0 International license [11].

Comparable data are not available for most other medium range scanners. Thus, with all our gathered experience with this scanner in the recent years, we are convinced that the RTC360 is a good solution for capturing reality within its limitations.

## Figures and Tables

**Table 1:** Specified 3D point accuracy and range noise of the RTC360 at different distances, with numerical values extracted from [12].

Target	Albedo	3D Point Accuracy (68% According to GUM)/ Range Noise of Single Measurement [mm]				
		**@ 5 m**	**@ 10 m**	**@ 20 m**	**@ 40 m**	**@ 60 m**
white	89%	1.4/0.3	1.9/0.4	2.9/0.5	5.3/0.6	7.8/1.0
gray	21%	1.5/0.4	2.0/0.5	3.2/0.6	5.7/0.8	8.2/2.0
black	8%	1.6/0.5	2.2/0.6	3.4/0.7	6.0/2.5	8.8/5.0

**Table 2:** Results of the determination of the absolute distance $d_{0}$.

EDM Target	$d_{0}$ [m]	$s_{d0}$ [mm]	Measured Points
Avery white	0.7727	0.06	24
Avery black	0.7726	0.06	24
Cardboard gray	0.7718	0.07	24
Cardboard black	0.7722	0.04	9

## Data Availability

The data presented in this study are openly available in TU Graz Repository at https://doi.org/10.3217/svnqw-54414.
